# Genomic Correlates of Virulence Attenuation in the Deadly Amphibian Chytrid Fungus, *Batrachochytrium dendrobatidis*

**DOI:** 10.1534/g3.115.021808

**Published:** 2015-09-01

**Authors:** Jeanine M. Refsnider, Thomas J. Poorten, Penny F. Langhammer, Patricia A. Burrowes, Erica Bree Rosenblum

**Affiliations:** * Department of Environmental Science, Policy, and Management, University of California, Berkeley, Berkeley, California 94720-3114; † School of Life Sciences, Arizona State University, Tempe, Arizona 85287; ‡ Department of Biology, University of Puerto Rico, San Juan, Puerto Rico 00931-3360

**Keywords:** chromosome copy number, chytridiomycosis, emerging infectious disease, gene expression, loss of heterozygosity

## Abstract

Emerging infectious diseasespose a significant threat to global health, but predicting disease outcomes for particular species can be complicated when pathogen virulence varies across space, time, or hosts. The pathogenic chytrid fungus *Batrachochytrium dendrobatidis* (*Bd*) has caused worldwide declines in frog populations. Not only do *Bd* isolates from wild populations vary in virulence, but virulence shifts can occur over short timescales when *Bd* is maintained in the laboratory. We leveraged changes in *Bd* virulence over multiple generations of passage to better understand mechanisms of pathogen virulence. We conducted whole-genome resequencing of two samples of the same *Bd* isolate, differing only in passage history, to identify genomic processes associated with virulence attenuation. The isolate with shorter passage history (and greater virulence) had greater chromosome copy numbers than the isolate maintained in culture for longer, suggesting that virulence attenuation may be associated with loss of chromosome copies. Our results suggest that genomic processes proposed as mechanisms for rapid evolution in *Bd* are correlated with virulence attenuation in laboratory culture within a single lineage of *Bd*. Moreover, these genomic processes can occur over extremely short timescales. On a practical level, our results underscore the importance of immediately cryo-archiving new *Bd* isolates and using fresh isolates, rather than samples cultured in the laboratory for long periods, for laboratory infection experiments. Finally, when attempting to predict disease outcomes for this ecologically important pathogen, it is critical to consider existing variation in virulence among isolates and the potential for shifts in virulence over short timescales.

Most infectious pathogens exhibit variation in virulence across space, time, or host species. For example, pathogens may affect hosts differentially (*e.g.*, Johnson *et al.* 2012), cause different outcomes under different environmental conditions (*e.g.*, Mitchell *et al.* 2005), and evolve increased or decreased virulence over time (*e.g.*, Boots *et al.* 2004). Understanding variation in pathogen virulence is essential to create accurate epidemiological models, predict disease outcomes, and understand mechanisms of pathogenicity (Jones *et al.* 2008).

*Batrachochytrium dendrobatidis* (*Bd*) is an ecologically important pathogen that provides an opportunity to understand mechanisms of variation in virulence. *Bd* is a chytrid fungus that attacks amphibian hosts and has led to population declines and extirpations around the world (*e.g.*, Berger *et al.* 1998; Bosch *et al.* 2001; Muths *et al.* 2003; Lips *et al.* 2006). However, there is dramatic variation in the outcome of *Bd* infection: some populations are decimated by *Bd* (*e.g.*, Schloegel *et al.* 2006; Crawford *et al.* 2010; Vredenburg *et al.* 2010), whereas others persist (Retallick *et al.* 2004; Woodhams *et al.* 2007; Lam *et al.* 2010). This variation in infection outcome is affected by three interacting factors: environmental conditions such as temperature (Voyles *et al.* 2012; Spitzen-Van der Sluijs *et al.* 2014), intrinsic differences in host susceptibility (*e.g.*, Bishop *et al.* 2009; Searle *et al.* 2011; Voyles *et al.* 2011; Ghal *et al.* 2012; Gervasi *et al.* 2013), and differences in virulence among *Bd* isolates (*e.g.*, Berger *et al.* 2005; Retallick and Miera 2005; Fisher *et al.* 2009; Farrer *et al.* 2011; Voyles 2011).

Here we focus on variation in virulence caused by intrinsic differences among *Bd* isolates. There is increasing evidence that *Bd* isolates differ in virulence even in controlled common garden conditions (*e.g.*, when hosts and environmental conditions are kept constant; Berger *et al.* 2005; Retallick and Miera 2005; Fisher *et al.* 2009; Farrer *et al.* 2011; Voyles 2011). Moreover, recent studies have revealed substantial genetic variation within *Bd* and dynamic genomic processes that could provide a mechanism for rapid shifts in virulence (Farrer *et al.* 2011, 2013; Rosenblum *et al.* 2013; Piovia-Scott *et al.* 2014).

An ideal framework for studying mechanisms of pathogen virulence is when shifts in virulence occur within a single pathogen lineage. More specifically, when virulence shifts can be induced in the laboratory, mechanisms of virulence can be examined directly without the confounding effects of spatial genetic structure, environmental variation, host-specific processes, or uncertain ancestry. Several recent studies have reported an attenuation of virulence in *Bd* over very short timescales in cultures maintained in the laboratory (Brem *et al.* 2013; Langhammer *et al.* 2013). Virulence attenuation also has been observed in other pathogenic fungi maintained in laboratory conditions (Lord and Roberts 1986; Butt *et al.* 2006; Safavi 2012; Janbon *et al.* 2014).

Here we make use of changes in *Bd* virulence that occurred in a laboratory setting to interrogate the mechanisms of variation in pathogen virulence. Specifically, we compared two *Bd* samples that originated from the same source but differed in passage history. One sample, JEL427-P9, was cryo-archived shortly after isolation (having undergone nine laboratory passages), and a second sample, JEL427-P39, was maintained in culture for 6 yr (39 laboratory passages). The two isolates differ in phenotypes relevant for virulence (*e.g.*, greater zoospore production rate in the JEL427-P9 isolate; Langhammer *et al.* 2013) as well as in their effect on frogs, with the JEL427-P9 isolate causing increased mortality (Langhammer *et al.* 2013). We undertook a genome resequencing study to identify genomic correlates of virulence attenuation and shed light on the genomic processes that may be important for the evolution of virulence in this host-pathogen system.

## Materials and Methods

### Culturing and DNA extraction

We extracted genomic DNA from two *Bd* isolates of strain JEL427 isolates that had undergone nine and 39 laboratory passages (JEL427-P9, the ancestral isolate, and JEL427-P39, the derived isolate, respectively). Before DNA extraction, we cultured each isolate for 5 d on two replicate 1% tryptone and 1% agar plates. We flooded the plates for zoospores and concentrated zoospores by using a tabletop centrifuge. We extracted genomic DNA using a phenol-chloroform protocol (Zolan and Pukkila 1986) modified for use in *Bd* (Joneson *et al.* 2011). Genomes from the two isolates were sequenced using the Illumina MiSeq platform (2 × 250 bp paired end reads) at the University of Idaho’s Genomics Resources Core Facility.

### Genome sequencing and SNP-calling

Sequence alignment and single-nucleotide polymorphism (SNP)-calling were performed as in Rosenblum *et al.* (2013). To summarize briefly, we used SeqyClean v. 1.8.10 (I. Y. Zhbannikov, S. S. Hunter, and M. L. Settles, SeqyClean: a Software Tool for Comprehensive Preprocessing of Sequence Data; https://github.com/ibest/seqyclean) to clean reads; remove polymerase chain reaction duplicates, contaminants, and adaptors; and trim sequences by quality scores. Reads were aligned to the reference genome of JEL423 (Broad Institute v. 17-Jan-2007) with the use of Bowtie 2 v. 2.1.0 (Langmead *et al.* 2009), and we used a best practices protocol for variant calling in GATK v. 1.4 (McKenna *et al.* 2010). We marked duplicate reads with Picard, and we realigned reads containing insertions/deletions (indels) with GATK walkers RealignerTargetCreator and IndelRealigner. We made final variant calls, and filtered false positives, using GATK UnifiedGenotyper and VariantFiltration walkers with the same filter parameter value as in Rosenblum *et al.* (2013). We then used snpEff software (version 2.0.5, Cingolani *et al.* 2012) and custom R scripts to extract summary information from the SNP dataset (*e.g.*, genomic position, synonymous *vs.* nonsynonymous mutations) to compare JEL427-P9 and JEL427-P39. We also used bedtools software (Quinlan and Hall 2010) to identify indels that occurred in coding exonic regions by using the gene feature annotation file downloaded from the Broad Institute’s online database (https://www.broadinstitute.org).

### Phylogenetic analysis

We inferred phylogenetic relationships among isolates by using the parsimony method described previously (Rosenblum *et al.* 2013). To summarize in brief, the SNPs were encoded to distinguish three character states (0−2): homozygous with respect to reference allele, heterozygous, and homozygous for the alternate allele. We performed 200 bootstrap replicates to generate node support values under the parsimony optimality criterion.

### Loss of heterozygosity analysis

We used a previously developed method to predict genomic regions affected by past “loss of heterozygosity” (LOH) events (Rosenblum *et al.* 2013), which result in large chromosomal regions that are homozygous. We implemented a hidden Markov model method to identify genomic regions with long stretches of homozygosity in the 15 largest supercontigs.

### Chromosome copy number variation

Variation in chromosome copy number is a change in the number of copies of a particular chromosome without a change in ploidy of the entire chromosome set. We estimated copy number for each supercontig, which are analogous to chromosomal segments in *Bd*, using SNP allele frequencies (as in Rosenblum *et al.* 2013). From the variant call format file, we extracted the mean allele frequency across SNPs for each supercontig. For each supercontig, we plotted the distribution of all SNP allele frequencies for that supercontig, and we used Gaussian kernel density in the R package KernSmooth to smooth the distribution. Each supercontig was then assigned a copy number based on the expected distribution of all SNP allele frequencies for a supercontig that was monosomic, disomic, trisomic, tetrasomic, or pentasomic. That is, allele frequencies would have a unimodal distribution centered at 0.5 for disomic chromosomes; a bimodal distribution with peaks at 0.33 and 0.67 for trisomic chromosomes; a trimodal distribution with peaks at 0.25, 0.5, and 0.75 for tetrasomic chromosomes; and a tetramodal distribution with peaks at 0.2, 0.4, 0.6, and 0.8 for pentasomic chromosomes.

### Analysis of gene expression

Finally, we evaluated the potential functional relevance of SNPs and indels using Gene Ontology (GO) categories and gene expression results from previous microarray experiments (Rosenblum *et al.* 2012). With the gene expression data, we asked whether sequence changes were more likely to occur in genes that were found previously to be up-regulated on frog skin. We hypothesized that, over the generations grown in culture (rather than on host tissue), selection on virulence-associated genes may be relaxed and could be evidenced by an elevated substitution rate relative to more highly constrained genes. To test this hypothesis for the SNP dataset, we conducted regression by implementing a generalized linear model assuming a negative binomial distribution of the number of nucleotide changes per gene in five differential expression categories: strong down-regulation, moderate down-regulation, no change, moderate up-regulation, and strong up-regulation. To test this hypothesis for the indel dataset, we used only three differential expression categories due to the relatively low number of indels in the dataset: down-regulation, no change, and up-regulation. We repeated the enrichment test with all indel-containing genes given the small number of indels. For all analyses, we included gene length as a covariate and generated models and estimated parameter 95% confidence intervals for both mutation types. For the SNPs, we partitioned the dataset into three types of nucleotide changes: nonsynonymous, synonymous, and combined. We also looked at the functional relevance of genes in our dataset that contained nonsynonymous mutations or indels and were up-regulated on frog skin in Rosenblum *et al.* (2012) by conducting an overrepresentation test of GO terms. We used the hypergeometric test in the GOstats R package (Falcon and Gentleman 2007) with the GO annotation assignments from Rosenblum *et al.* (2013) and filtered the enrichment results to exclude enriched GO terms that contained only one gene in the gene list.

### Data availability

Genomic sequence data are accessioned in the National Center for Biotechnology Information Short Read Archive (accession # SRP 049423)

## Results

**Figure 1:**
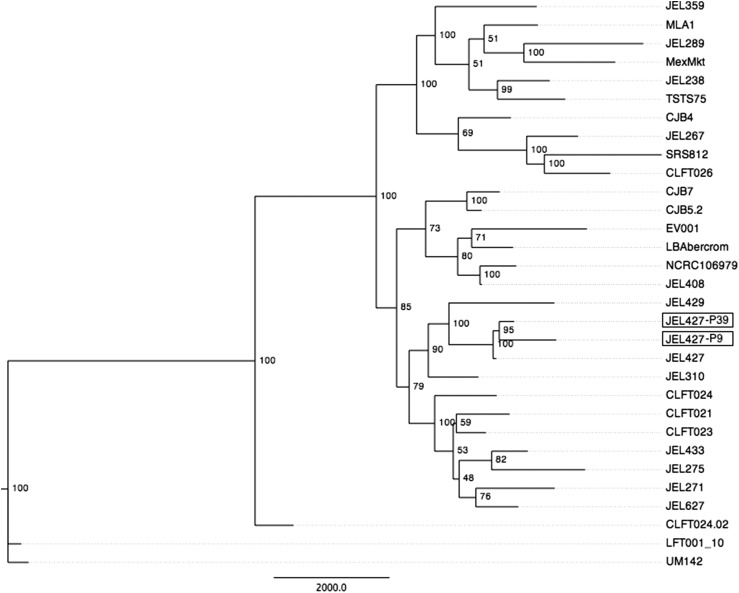
Rooted *Batrachochytrium dendrobatidis* phylogeny based on genomic data. Maximum parsimony tree is shown with nodal support values generated from 200 bootstrap replicates. The two JEL427 isolates (highlighted with boxes) cluster together and are solidly nested within the Global Panzootic Lineage (GPL). The isolate labeled “JEL427” was previously sequenced in Rosenblum *et al.* (2013).

We obtained a depth of coverage for aligned reads of 33X and 20X for the JEL427-P9 and JEL427-P39 isolates, respectively. Using a phylogeny that included 29 isolates from a previous study (Rosenblum *et al.* 2013), we found that the two JEL427 isolates clustered together with high bootstrap support and nested within the most recently derived and widely distributed clade, the Global Panzootic Lineage (GPL; Figure 1). We note that the terminal branch-lengths of the JEL427 isolates had a *prima facie* counterintuitive pattern. This pattern is likely explained by the trend of genotype changes in identified LOH regions. For LOH regions across the genome, the ancestral isolate (*i.e.*, JEL427-P9) tended to contain the greatest density of heterozygous sites, and the derived isolate (*i.e.*, JEL427-P39) showed a loss of allelic diversity. This trend would lead to the branch-length pattern seen in Figure 1, where JEL427-P9 contains the longer terminal branch.

**Figure 2:**
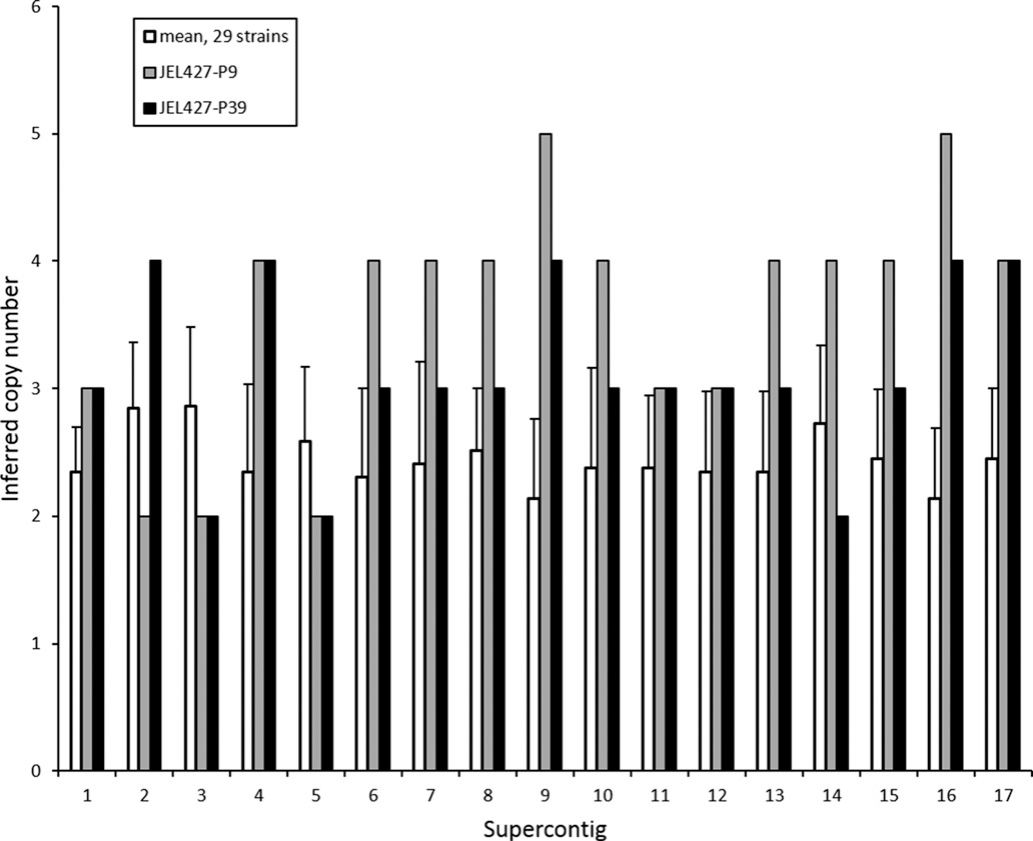
Evidence for decrease in chromosomal copy numbers between the ancestral *Batrachochytrium dendrobatidis* (*Bd*) isolate (JEL427-P9) and the derived isolate (JEL427-P39), which differ only in laboratory passage history. Mean copy numbers from 29 *Bd* isolates resequenced in Rosenblum *et al.* (2013) are shown for comparison. Supercontigs are analogous to chromosomes in *Bd*.

The most compelling genomic patterns we observed were chromosomal copy number variation in the ancestral isolate. Chromosomal copy number for the JEL427-P39 isolate varied from disomic to tetrasomic, while the JEL427-P9 isolate varied from disomic to pentasomic. Most notably, the JEL427-P39 isolate showed a decrease in copy number for 9 of 17 supercontigs compared with the JEL427-P9 isolate (Figure 2). Specifically, supercontigs 6, 7, 8, 9, 10, 13, 14, 15, and 16 showed a greater chromosome copy number in the JEL427-P9 isolate compared with the JEL427-P39 isolate, and when compared to the average of other isolates studied to date (Rosenblum *et al.* 2013; Figure 2).

After SNP filtering, we observed 2,231 changes between JEL427-P9 and JEL427-P39 (Supporting Information, Table S1). The snpEff results were qualitatively similar to the pattern observed in the Rosenblum *et al.* (2013) dataset of 29 global isolates. Roughly half of the SNPs occurred in regions either up- or down-stream of genes (within 1 kb). In coding regions, ∼9% of all SNPs caused nonsynonymous and ∼20% caused synonymous changes, and ∼18% of SNPs occurred in introns. Overall, the SNPs were distributed across supercontigs in a similar pattern for both comparisons. However, a few supercontigs (*i.e.*, supercontigs 7, 14, and 17) contained an elevated number of SNPs. Our indel analysis uncovered 470 indels between JEL427-P9 and JEL427-P39, 149 of which occurred in coding exonic regions.

To determine whether LOH patterns affected the rate of nucleotide changes, we calculated the synonymous change rate in LOH and non-LOH regions. LOH patterns did not appear to have a major effect on genotype change, as the synonymous change rates were 0.00102 and 0.00105 in LOH and non-LOH regions, respectively.

**Figure 3:**
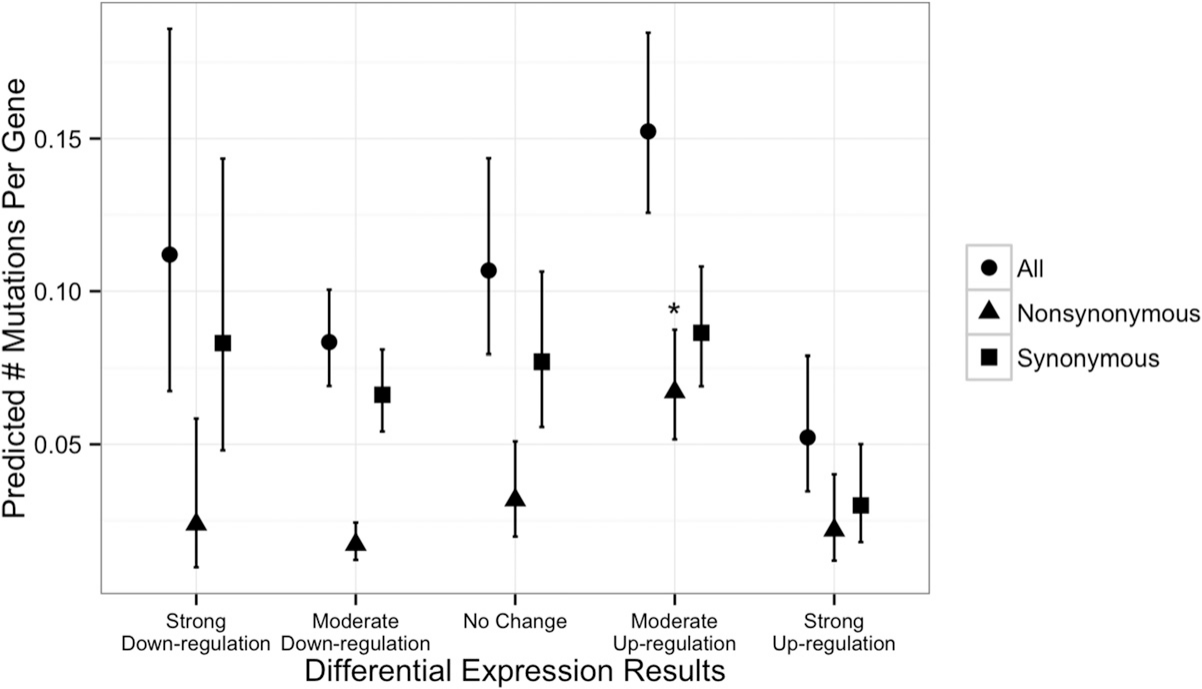
*Batrachochytrium dendrobatidis* genes that were moderately up-regulated in the presence of frog skin showed greater-than-expected frequency of nonsynonymous mutations in the JEL427-P39 (compared with the JEL427-P9) isolate. The x-axis clusters genes based on their differential expression profiles in a previous study (Rosenblum *et al.* 2012), where “up-regulation” implies increased gene expression when grown on frog skin compared to standard tryptone growth medium. The y-axis shows number of mutations at all sites (circles), nonsynonymous sites (triangles), and synonymous sites (squares). The asterisk indicates that genes that were moderately up-regulated in frog skin had a significantly higher number of nonsynonymous mutational changes.

The analysis including previous gene expression results (from Rosenblum *et al.* 2012) suggested that the differential expression (on frog skin) coefficient of a gene was significantly correlated with the number of nucleotide changes observed in this study. Genes that were moderately up-regulated when grown on frog skin had a greater number of nonsynonymous changes compared with genes without differential expression in different growth conditions (Figure 3, P = 0.00648 in negative binomial regression analysis). In all, 84 genes met the two criteria of being up-regulated and containing ≥1 nonsynonymous change. The hypergeometric test for overrepresentation of GO terms did not reveal any notable patterns (Table S2), which was likely due to the relatively low number of annotated genes in the gene list. However, the gene set contained one M36 metallopeptidase gene, a gene family that previously has been hypothesized to play a role in *Bd* virulence (Joneson *et al.* 2011).

**Figure 4:**
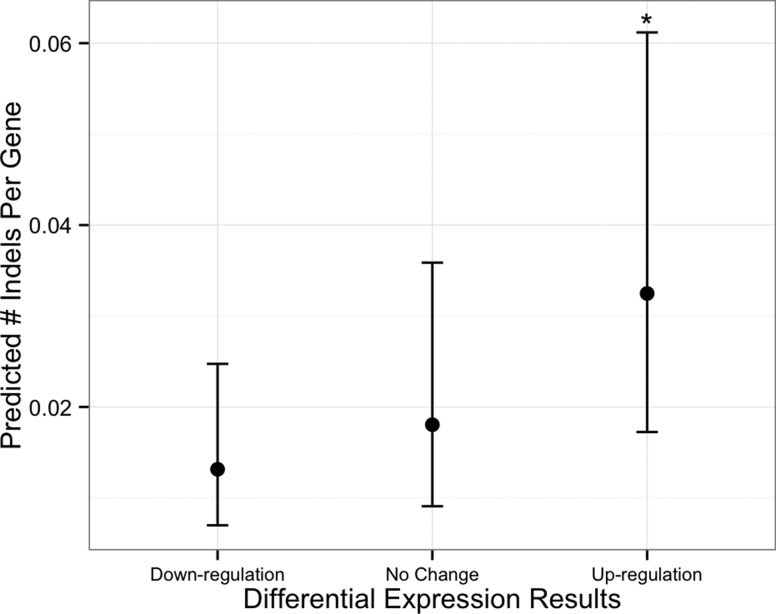
*Batrachochytrium dendrobatidis* genes that were up-regulated in the presence of frog skin showed greater-than-expected frequency of indels in the JEL427-P39 (compared with the JEL427-P9) isolate. The x-axis clusters genes based on their differential expression profiles in a previous study (Rosenblum *et al.* 2012), where “up-regulation” implies increased gene expression when grown on frog skin compared to standard tryptone growth medium. The y-axis shows number of indels. The asterisk indicates the genes that were up-regulated in frog skin had a significantly greater number of indels.

In the gene expression analysis with indels, there was a weak significant effect of increased gene expression level on indel occurrence (Figure 4, P = 0.048). We found a significant enrichment of GO terms “proteolysis” and “protein metabolic process” in the gene list of genes with increased expression and indels; however, we note that there were only two genes in the proteolysis gene set and three genes in the protein metabolic process gene set (*P* = 0.029 for both GO terms). For the analysis including all indel-containing genes (regardless of expression), we found the list of genes containing exonic indels was overenriched for the GO terms related to proteolysis and metabolism (Table 1).

**Table 1:** Overenriched Gene Ontology (GO) terms in genes containing exonic indels

GO Category	GO Term	*P* value	Count	GO Term Size	GO Term Name
Biological process	GO:0006508	0.0010	9	282	Proteolysis
	GO:0043170	0.0296	18	1290	Macromolecule metabolic process
	GO:0008152	0.0431	25	2147	Metabolic process
Molecular function	GO:0008236	0.0035	4	64	Serine-type peptidase activity
	GO:0016787	0.0104	17	991	Hydrolase activity
	GO:0004190	0.0267	4	115	Aspartic-type endopeptidase activity
	GO:0070011	0.0435	5	214	Peptidase activity, acting on L-amino acid peptides

## Discussion

Determining how quickly pathogens can exhibit shifts in virulence, and the mechanisms underlying virulence shifts, is critical for understanding and mitigating emerging infectious disease threats. Our study focused on identifying the genomic correlates of rapid virulence shifts within a single lineage of the pathogenic fungus, *Bd*.

Studying the evolution of genomic mechanisms within individual *Bd* isolates is key to understanding variation in pathogen virulence. Our genomic data placed the two JEL427 isolates together and solidly within the GPL (Figure 1), the *Bd* clade that is most recent evolutionarily and the most widespread geographically. Previous studies have demonstrated substantial genetic variation and the potential for rapid genomic evolution within the GPL (Rosenblum *et al.* 2013; Farrer *et al.* 2013). The genetic variation identified within the GPL may reflect functional variation, and indeed the *Bd* isolates used in our study show differences in virulence-related phenotypes that accumulated rapidly, over only 30 generations (∼6 yr, Langhammer *et al.* 2013). The isolate with a longer passage history (JEL427-P39) was less virulent compared with the ancestral isolate that was cryo-archived immediately on isolation (JEL427-P9; Langhammer *et al.* 2013). Specifically, JEL427-P9 produced zoospores at a greater rate and caused increased mortality of frogs compared with JEL427-P39 (Langhammer *et al.* 2013). Studying a rapid virulence shift within a single lineage controls for many confounding factors common in studies of pathogens in natural systems (*e.g.*, environmental conditions, host susceptibility, and pathogen ancestry) and provides an opportunity to assess the genomic changes associated with attenuation of virulence. We therefore used our whole-genome resequencing data to assess whether specific genomic mechanisms were associated with observed virulence attenuation.

Lability in chromosomal copy number and heterozygosity have been identified as genomic process that could lead to rapid evolution in *Bd* (Rosenblum *et al.* 2013; Farrer *et al.* 2013). We therefore tested for differences in chromosome copy number and LOH between *Bd* isolates differing in laboratory passage history. We did not find evidence that LOH was associated with attenuation of virulence, but we did find striking changes in chromosomal copy number over the short timescale studied here (Figure 2). Specifically, we found that the derived isolate JEL427-P39, which was maintained in culture for ∼6 yr, had, in general, fewer copies of chromosomes than the ancestral isolate JEL427-P9. Decreased chromosomal copy number in the less-virulent isolate with a longer passage history was a highly consistent pattern, observed in more than half of the analyzed chromosomal segments (*i.e.*, 9 of the 17 largest super-contigs). The complementary pattern of greater chromosome copy number in highly virulent *Bd* isolates in nature also has been observed (Piovia-Scott *et al.* 2014), suggesting that copy number variation is potentially an important mechanism for rapid changes in virulence more generally in this pathogen. Although an increase in chromosome copy number can lead to increased virulence, possibly because it results in more copies of genes related to pathogenicity, such a process may entail costs to the pathogen. Therefore, in laboratory culture media (*i.e.*, without natural selection imposed by a host), loss of chromosomal copies may occur in a predictable manner. Attenuation of virulence has been associated with chromosomal changes in other fungal pathogens, for example, losses of minichromosomes containing genes related to pathogenicity in fungal pathogens of insects and plants (Wang *et al.* 2002, 2003; Akamatsu *et al.* 1999), and karyotype changes and loss of chromosome segments in *Cryptococcus*, a fungal pathogen of humans (Franzot *et al.* 1998; Janbon *et al.* 2014). Thus, our observation that virulence attenuation was associated with loss of chromosome copies may reflect a fairly general pattern.

Results regarding copy number changes from another recent study of *Bd* genomics bear specific mention here. Farrer *et al.* (2013) also compared replicate lines of a *Bd* isolate, which were passaged in the laboratory for 40 generations with and without exposure to amphibian antimicrobial peptides. In contrast to our results, Farrer *et al.* (2013) found fewer changes in chromosome copy number in their isolates after 40 generations (*i.e.*, their control isolate lost a copy of supercontig 4 and gained a copy of supercontig 5 and their isolate passaged with antimicrobial peptides gained a copy of supercontig 5). However, we note two differences between the studies that may explain why we observed more changes in chromosomal copy number. First the isolates were derived from two divergent lineages of *Bd* (*Bd*GPL in our study and *Bd*CH in Farrer *et al.* 2013), which may exhibit different dynamics of genome evolution. Second, the more ancestral isolate in our study (JEL427-P9) contains greater chromosome copy numbers than the ancestral isolate (ACON) in Farrer *et al.* (2013): mean copy number 3.5 *vs.* 2.8, respectively, for supercontigs 1−15. The greater starting chromosome copy number in our study may have made the isolate more prone to losing chromosome copies. These hypotheses can be tested with formal meta-analyses as additional laboratory passage experiments are conducted with *Bd*. Specifically laboratory passage experiments with different isolates under different selection regimes are needed, particularly those that characterize virulence phenotypes and genomic changes over time.

We also tested for the enrichment of mutations (SNPs and indels) in genes with putative virulence effects. We found that the list of indel-bearing genes contained an overenrichment for GO term annotations related to proteolysis and metabolism (Table 1). The occurrence of indels in protease genes was particularly notable, given the hypothesized role of proteases in *Bd* virulence (Joneson *et al.* 2011; Rosenblum *et al.* 2013). Given that indels are likely to have deleterious effects on protein production and function, the occurrence of indels may be indicative of loss-of-function effects for such genes. This supposition requires further testing but is consistent with the hypothesis that the laboratory-culturing environment relaxes selection on virulence-related genes relative to the host environment. With regard to SNPs in our dataset, we found that *Bd* genes previously demonstrated to be up-regulated when grown in frog skin (Rosenblum *et al.* 2012) had a greater frequency of mutational changes in the two JEL427 isolates than genes not up-regulated in frog skin (Figure 3). In particular, one of the genes that was both up-regulated in the presence of frog skin and contained at least one nonsynonymous change between the JEL427-P9 and JEL427-P39 samples was a M36 metallopeptidase, a gene family that has been hypothesized previously to play a role in *Bd* virulence (Joneson *et al.* 2011; Rosenblum *et al.* 2013).

Here we show that the genomic processes previously proposed as mechanisms for rapid evolution in *Bd* are correlated with attenuation of virulence in laboratory culture within a single lineage of *Bd*. The fact that virulence attenuates *in vitro* suggests that maintaining virulence genes and extra copies of chromosomes may be costly to *Bd* at the cellular level. Moreover, the speed with which shifts in virulence can apparently occur in *Bd* suggests that this pathogen has a considerable capacity to respond to novel conditions. Over 6 yr of laboratory passage, we observed 0.096 changes per kilobase, which translates into a mutation rate of 1.6 × 10^−5^ changes per site per year (Table S1). A mutation rate of this magnitude is high compared with other fungi (*e.g.*, *Saccharomyces cerevisiae*; Zeyl and Devisser 2001) but similar to other fungal pathogens such as *Cryptococcus neoformans* (Xu 2002) and *Mycosphaerella graminicola* (Stukenbrock *et al.* 2006). Future work should endeavor to identify proximate triggers of rapid genomic changes in laboratory settings and investigate dynamics of genomic evolution of *Bd* in nature. From a practical standpoint, our study suggests that controlled infection experiments designed to predict disease outcomes should use recently isolated samples, rather than samples cultured in the laboratory for long periods. In addition, researchers should cryo-archive *Bd* samples immediately after isolation. *Bd* has a demonstrated ability to respond quickly to novel hosts and environmental conditions through relatively rapid increases or decreases in virulence, which has likely contributed to its spread around the world. The results of our study emphasize the importance of considering virulence on an isolate-specific basis when creating epidemiological models and predicting disease outcomes for this ecologically important pathogen.

## Supplementary Material

Supporting Information
