# Supplementary material for: Genomic Correlates of Virulence Attenuation in the Deadly Amphibian Chytrid Fungus, Batrachochytrium dendrobatidis
Source: G3 (Bethesda). 2015 Sep 1;5(11):2291–8. doi: 10.1534/g3.115.021808 (PMC4632049; doi:10.1534/g3.115.021808)
Supplement: Supporting Information [file supp_5_11_2291__index.html]

Genomic Correlates of Virulence Attenuation in the Deadly Amphibian Chytrid Fungus, Batrachochytrium dendrobatidis — Supporting Information 

# Genomic Correlates of Virulence Attenuation in the Deadly Amphibian Chytrid Fungus, *Batrachochytrium dendrobatidis*

## Supporting Information for Refsnider *et al.*, 2015

**Files in this Data Supplement:**

- Supporting Information - Tables S1-S2 (PDF, 200 KB)
- Table S1 - (A) Summary of 2,231 nucleotide changes between two *Bd* isolates, JEL427-P9 and JEL427-P39, differing only in laboratory passage history. (B) Results from Rosenblum *et al.* 2013 (Proc. Natl. Acad. Sci.) (PDF, 149 KB)
- Table S2 - Summary of potential functional relevance of nucleotide changes between two *Bd* isolates, JEL427-P9 and JEL427-P39, differing only in laboratory passage history. (PDF, 137 KB)
