# Supplementary material for: Genomic Correlates of Virulence Attenuation in the Deadly Amphibian Chytrid Fungus, Batrachochytrium dendrobatidis
Source: G3 (Bethesda). 2015 Sep 1;5(11):2291–8. doi: 10.1534/g3.115.021808 (PMC4632049; doi:10.1534/g3.115.021808)
Supplement: Supporting Information [file supp_g3.115.021808_021808SI.pdf]

G3 · Genes | Genomes | Genetics

Genomic correlates of virulence attenuation in the deadly amphibian chytrid fungus, *Batrachochytrium dendrobatidis*

Supporting Information

Jeanine M. Refsnider*, Thomas J. Poorten*, Penny F. Langhammer§, Patricia A. Burrowes†, and Erica Bree Rosenblum*

*Department of Environmental Science, Policy, and Management; University of California, Berkeley; Berkeley, CA 94720-3114

§School of Life Sciences; Arizona State University; Tempe, AZ 85287

†Department of Biology; University of Puerto Rico; San Juan, Puerto Rico

Genomic sequence data are accessioned in the National Center for Biotechnology Information Short Read Archive (accession # SRP049423).

Corresponding author:
Erica Bree Rosenblum
Department of Environmental Science, Policy, and Management
University of California, Berkeley
Berkeley, CA 94720-3114 U.S.A.
rosenblum@berkeley.edu

DOI: 10.1534/g3.115.021808

Table S1 Summary of 2,231 nucleotide changes between two Bd isolates, JEL427-P9 and JEL427-P39, differing only in laboratory passage history (A). Following SNP filtering, summary information from the SNP dataset (e.g., genomic position, synonymous vs. nonsynonymous mutations) were extracted using snpEff software (version 2.0.5, Cingolani et al. 2012) and custom R scripts to compare the two isolates. The snpEff results were qualitatively similar to the pattern observed in the Rosenblum et al. 2013 dataset of 29 global isolates (B).

A: JEL427-P9 to JEL427-P39

Changes by type

	# of changes	Per cent of changes
**DOWNSTREAM**	894	25.88%
**INTERGENIC**	17	0.49%
**INTRON**	641	18.56%
**NON_SYNONYMOUS_CODING**	300	8.69%
**SPLICE_SITE_ACCEPTOR**	1	0.03%
**SPLICE_SITE_DONOR**	1	0.03%
**START_GAINED**	1	0.03%
**STOP_GAINED**	2	0.06%
**STOP_LOST**	0	0.00%
**SYNONYMOUS_CODING**	642	18.59%
**SYNONYMOUS_STOP**	2	0.06%
**UPSTREAM**	930	26.93%
**UTR_3_PRIME**	17	0.49%
**UTR_5_PRIME**	6	0.17%

Chromosome	Length	Changes	Change rate	Change per kb	Normalized
bden_JEL423_supercont1.1	4,440,149	503	8,827	0.113	0.051
bden_JEL423_supercont1.2	2,313,122	31	74,616	0.013	0.006

bden_JEL423_supercont1.3	1,829,408	62	29,506	0.034	0.015
bden_JEL423_supercont1.4	1,803,316	160	11,270	0.089	0.040
bden_JEL423_supercont1.5	1,707,251	42	40,648	0.025	0.011
bden_JEL423_supercont1.6	1,545,501	194	7,966	0.126	0.056
bden_JEL423_supercont1.7	1,398,854	263	5,318	0.188	0.084
bden_JEL423_supercont1.8	1,069,847	49	21,833	0.046	0.021
bden_JEL423_supercont1.9	1,057,463	123	8,597	0.116	0.052
bden_JEL423_supercont1.10	1,012,305	137	7,389	0.135	0.061
bden_JEL423_supercont1.11	979,369	58	16,885	0.059	0.027
bden_JEL423_supercont1.12	937,107	150	6,247	0.160	0.072
bden_JEL423_supercont1.13	898,261	158	5,685	0.176	0.079
bden_JEL423_supercont1.14	857,155	89	9,630	0.104	0.047
bden_JEL423_supercont1.15	557,602	100	5,576	0.179	0.080
bden_JEL423_supercont1.16	498,254	57	8,741	0.114	0.051
bden_JEL423_supercont1.17	243,426	50	4,868	0.205	0.092
bden_JEL423_supercont1.21	28,371	4	7,092	0.141	0.063
bden_JEL423_supercont1.46	7,014	1	7,014	0.143	0.064
**Total**	**23,183,77**	**2,231**	**10,391**	**0.096**	
**Ts/Tv ratio**	**5.4109**				

<u>Genotype changes</u>

	#	proportion
het -> hom	646	0.299
hom -> het	1477	0.683
hom1 -> hom2	38	0.018
**Total**	**2161**	

B: Results from Rosenblum *et al*. 2013 (Proc. Natl. Acad. Sci.)

Changes by type

	# of changes	Per cent of changes
**DOWNSTREAM**	49,685	26.59%
**INTERGENIC**	1,970	1.05%
**INTRON**	26,688	14.28%
**NON_SYNONYMOUS_CODING**	19,756	10.57%
**SPLICE_SITE_ACCEPTOR**	59	0.03%
**SPLICE_SITE_DONOR**	75	0.04%
**START_GAINED**	50	0.03%
**STOP_GAINED**	114	0.06%
**STOP_LOST**	26	0.01%
**SYNONYMOUS_CODING**	38,397	20.55%
**SYNONYMOUS_STOP**	51	0.03%
**UPSTREAM**	48,665	26.05%
**UTR_3_PRIME**	973	0.52%
**UTR_5_PRIME**	306	0.16%

Chromosome	Length	Changes	Change rate	Change per kb	Normalized
bden_JEL423_supercont1.1	4,440,149	23,541	188	5.302	0.043
bden_JEL423_supercont1.2	2,313,122	10,157	227	4.391	0.036
bden_JEL423_supercont1.3	1,829,408	9,588	190	5.241	0.043
bden_JEL423_supercont1.4	1,803,316	9,178	196	5.090	0.042
bden_JEL423_supercont1.5	1,707,251	8,741	195	5.120	0.042
bden_JEL423_supercont1.6	1,545,501	8,008	192	5.181	0.042
bden_JEL423_supercont1.7	1,398,854	7,764	180	5.550	0.045
bden_JEL423_supercont1.8	1,069,847	5,834	183	5.453	0.045
bden_JEL423_supercont1.9	1,057,463	6,080	173	5.750	0.047
bden_JEL423_supercont1.10	1,012,305	5,766	175	5.696	0.047
bden_JEL423_supercont1.11	979,369	5,427	180	5.541	0.045
bden_JEL423_supercont1.12	937,107	4,907	190	5.236	0.043

bden_JEL423_supercont1.13	898,261	5,433	165	6.048	0.049
bden_JEL423_supercont1.14	857,155	3,102	276	3.619	0.030
bden_JEL423_supercont1.15	557,602	4,124	135	7.396	0.061
bden_JEL423_supercont1.16	498,254	2,788	178	5.596	0.046
bden_JEL423_supercont1.17	243,426	1,333	182	5.476	0.045
bden_JEL423_supercont1.18	48,566	50	971	1.030	0.008
bden_JEL423_supercont1.19	69,118	118	585	1.707	0.014
bden_JEL423_supercont1.20	45,066	59	763	1.309	0.011
bden_JEL423_supercont1.21	28,371	222	127	7.825	0.064
**Total**	**23,339,511**	**122,220**		**5.237**	

Table S2 Summary of potential functional relevance of nucleotide changes between two *Bd* isolates, JEL427-P9 and JEL427-P39, differing only in laboratory passage history. We assessed potential functional relevance of genes that had at least one nonsynonymous mutation in our dataset and were up-regulated on frog skin in Rosenblum *et al*. (2012) by conducting an over-representation test of Gene Ontology (GO) terms. We used the hypergeometric test in the GOstats R package (Falcon & Gentleman 2007) with the GO annotation assignments from Rosenblum *et al*. (2013). In all, 84 genes were both up-regulated and contained ≥1 nonsynonymous change. The hypergeometric test for over-representation of GO terms did not reveal any notable patterns, although the gene set contained one M36 metallopeptidase gene, a gene family that has previously been hypothesized to play a role in *Bd* virulence (Joneson *et al*. 2011).

GOBPID	P-value	Odds ratio	ExpCount	Count	Size	Term	Gene Ids
GO:0032313	0.006	21.595	0.116	2	20	regulation of Rab GTPase activity	BDEG_00189;BDEG_00594
GO:0006369	0.006	Inf	0.006	1	1	termination of RNA polymerase II transcription	BDEG_05286
GO:0043144	0.006	Inf	0.006	1	1	snoRNA processing	BDEG_05286
GO:0016180	0.006	Inf	0.006	1	1	snRNA processing	BDEG_05286
GO:0043087	0.009	16.870	0.145	2	25	regulation of GTPase activity	BDEG_00189;BDEG_00594
GO:0033121	0.009	16.870	0.145	2	25	regulation of purine nucleotide catabolic process	BDEG_00189;BDEG_00594
GO:0009118	0.009	16.870	0.145	2	25	regulation of nucleoside metabolic process	BDEG_00189;BDEG_00594
GO:0006140	0.009	16.161	0.151	2	26	regulation of nucleotide metabolic process	BDEG_00189;BDEG_00594
GO:0031329	0.009	16.161	0.151	2	26	regulation of cellular catabolic process	BDEG_00189;BDEG_00594
GO:0006821	0.012	182.533	0.012	1	2	chloride transport	BDEG_05538
GO:0048583	0.014	7.034	0.523	3	90	regulation of response to stimulus	BDEG_00189;BDEG_00452;BDEG_00594
GO:0010646	0.014	7.034	0.523	3	90	regulation of cell communication	BDEG_00189;BDEG_00452;BDEG_00594
GO:0023051	0.014	7.034	0.523	3	90	regulation of signaling	BDEG_00189;BDEG_00452;BDEG_00594
GO:0006303	0.017	91.233	0.017	1	3	double-strand break repair via nonhomologous end joining	BDEG_06507
GO:0016575	0.017	91.233	0.017	1	3	histone deacetylation	BDEG_00020
GO:0006012	0.017	91.233	0.017	1	3	galactose metabolic process	BDEG_04530
GO:0006807	0.018	3.247	4.565	9	786	nitrogen compound metabolic process	BDEG_00189;BDEG_00252;BDEG_00594;BDEG_03087; BDEG_03599;BDEG_04834;BDEG_05203;BDEG_05286;BDEG_06507
GO:0050790	0.027	8.957	0.261	2	45	regulation of catalytic activity	BDEG_00189;BDEG_00594
GO:0051174	0.027	8.957	0.261	2	45	regulation of phosphorus metabolic process	BDEG_00189;BDEG_00594
GO:0046578	0.032	8.182	0.285	2	49	regulation of Ras protein signal transduction	BDEG_00189;BDEG_00594
GO:0016569	0.034	36.453	0.035	1	6	covalent chromatin modification	BDEG_00020

GO:0035601	0.040	30.367	0.041	1	7	protein deacylation	BDEG_00020
GO:0052803	0.046	26.019	0.046	1	8	imidazole-containing compound metabolic process	BDEG_04834
GO:0000105	0.046	26.019	0.046	1	8	histidine biosynthetic process	BDEG_04834
GO:0006184	0.053	6.068	0.377	2	65	GTP catabolic process	BDEG_00189;BDEG_00594
GO:0006139	0.056	3.452	1.530	4	309	nucleobase-containing compound metabolic process	BDEG_03087;BDEG_03599;BDEG_05203;BDEG_06507
GO:0009146	0.056	5.877	0.389	2	67	purine nucleoside triphosphate catabolic process	BDEG_00189;BDEG_00594
GO:0009261	0.056	5.877	0.389	2	67	ribonucleotide catabolic process	BDEG_00189;BDEG_00594
GO:0009203	0.056	5.877	0.389	2	67	ribonucleoside triphosphate catabolic process	BDEG_00189;BDEG_00594
GO:0046130	0.056	5.877	0.389	2	67	purine ribonucleoside catabolic process	BDEG_00189;BDEG_00594
GO:0072523	0.056	5.877	0.389	2	67	purine-containing compound catabolic process	BDEG_00189;BDEG_00594
GO:0009164	0.058	5.786	0.395	2	68	nucleoside catabolic process	BDEG_00189;BDEG_00594
GO:1901292	0.062	5.528	0.412	2	71	nucleoside phosphate catabolic process	BDEG_00189;BDEG_00594
GO:1902531	0.062	5.528	0.412	2	71	regulation of intracellular signal transduction	BDEG_00189;BDEG_00594
GO:1901068	0.062	5.528	0.412	2	71	guanosine-containing compound metabolic process	BDEG_00189;BDEG_00594
GO:0065007	0.063	2.788	2.259	5	389	biological regulation	BDEG_00189;BDEG_00252;BDEG_00382;BDEG_00452; BDEG_00594
GO:0006357	0.084	12.976	0.087	1	15	regulation of transcription from RNA polymerase II promoter	BDEG_00252
GO:0006364	0.089	12.107	0.093	1	16	rRNA processing	BDEG_05286
GO:0009968	0.100	10.675	0.105	1	18	negative regulation of signal transduction	BDEG_00452
GO:0038032	0.100	10.675	0.105	1	18	termination of G-protein coupled receptor signaling pathway	BDEG_00452
GO:0008277	0.100	10.675	0.105	1	18	regulation of G-protein coupled receptor protein signaling pathway	BDEG_00452
GO:0009205	0.103	4.065	0.552	2	95	purine ribonucleoside triphosphate metabolic process	BDEG_00189;BDEG_00594
GO:0009141	0.105	4.020	0.558	2	96	nucleoside triphosphate metabolic process	BDEG_00189;BDEG_00594
GO:0006325	0.116	9.063	0.122	1	21	chromatin organization	BDEG_00020
GO:0042278	0.120	3.693	0.604	2	104	purine nucleoside metabolic process	BDEG_00189;BDEG_00594
GO:0005996	0.121	8.629	0.128	1	22	monosaccharide metabolic process	BDEG_04530
GO:0009150	0.128	3.549	0.627	2	108	purine ribonucleotide metabolic process	BDEG_00189;BDEG_00594
GO:0009119	0.130	3.514	0.633	2	109	ribonucleoside metabolic process	BDEG_00189;BDEG_00594
GO:0006820	0.131	7.872	0.139	1	24	anion transport	BDEG_05538
GO:0019693	0.132	3.480	0.639	2	110	ribose phosphate metabolic process	BDEG_00189;BDEG_00594
GO:0048523	0.141	7.237	0.151	1	26	negative regulation of cellular process	BDEG_00452
GO:0008033	0.146	6.956	0.157	1	27	tRNA processing	BDEG_05286

GO:1901657	0.148	3.230	0.685	2	118	glycosyl compound metabolic process	BDEG_00189;BDEG_00594
GO:0022613	0.176	5.640	0.192	1	33	ribonucleoprotein complex biogenesis	BDEG_05286
GO:0007166	0.190	5.150	0.209	1	36	cell surface receptor signaling pathway	BDEG_00452
GO:0071704	0.194	2.110	7.972	10	1481	organic substance metabolic process	BDEG_00020;BDEG_00252;BDEG_03087;BDEG_03599;BDEG_04530; BDEG_04834;BDEG_05033;BDEG_05203;BDEG_05286;BDEG_06507
GO:0071840	0.205	2.593	0.842	2	145	cellular component organization or biogenesis	BDEG_00020;BDEG_05286
GO:0006351	0.235	2.349	0.923	2	159	transcription, DNA-templated	BDEG_00252;BDEG_05286
GO:1902589	0.237	3.991	0.267	1	46	single-organism organelle organization	BDEG_00020
GO:0006281	0.241	3.903	0.273	1	47	DNA repair	BDEG_06507
GO:0033554	0.250	3.738	0.285	1	49	cellular response to stress	BDEG_06507
GO:0055086	0.261	2.172	0.993	2	171	nucleobase-containing small molecule metabolic process	BDEG_00189;BDEG_00594
GO:1901575	0.286	2.031	1.057	2	182	organic substance catabolic process	BDEG_00189;BDEG_00594
GO:0051716	0.286	2.037	1.059	2	191	cellular response to stimulus	BDEG_00382;BDEG_00452
GO:0023052	0.306	1.927	1.109	2	191	signaling	BDEG_00382;BDEG_00452
GO:0008652	0.314	2.832	0.372	1	64	cellular amino acid biosynthetic process	BDEG_04834
GO:0044237	0.322	1.449	8.566	10	1475	cellular metabolic process	BDEG_00020;BDEG_00189;BDEG_00252;BDEG_00594;BDEG_03087; BDEG_03599;BDEG_04834;BDEG_05203;BDEG_05286;BDEG_06507
GO:1901605	0.326	2.700	0.389	1	67	alpha-amino acid metabolic process	BDEG_04834
GO:0034660	0.334	2.619	0.401	1	69	ncRNA metabolic process	BDEG_05286
GO:0035556	0.347	2.497	0.419	1	71	intracellular signal transduction	BDEG_00382
GO:0044710	0.377	1.502	2.183	3	451	single-organism metabolic process	BDEG_00020;BDEG_04530;BDEG_04834
GO:0050794	0.385	1.619	1.316	2	269	regulation of cellular process	BDEG_00252;BDEG_00382
GO:0034654	0.391	1.573	1.336	2	230	nucleobase-containing compound biosynthetic process	BDEG_00252;BDEG_05286
GO:0016053	0.399	2.082	0.499	1	86	organic acid biosynthetic process	BDEG_04834
GO:0006259	0.429	1.891	0.548	1	99	DNA metabolic process	BDEG_03087
GO:0044763	0.434	1.311	3.316	4	689	single-organism cellular process	BDEG_00382;BDEG_00452;BDEG_04834;BDEG_05538
GO:0019219	0.439	1.839	0.564	1	110	regulation of nucleobase-containing compound metabolic process	BDEG_00252
GO:2001141	0.474	1.640	0.627	1	108	regulation of RNA biosynthetic process	BDEG_00252
GO:0006396	0.477	1.624	0.633	1	109	RNA processing	BDEG_05286
GO:0006629	0.486	1.578	0.650	1	112	lipid metabolic process	BDEG_00382
GO:0009889	0.492	1.549	0.662	1	114	regulation of biosynthetic process	BDEG_00252
GO:2000112	0.492	1.549	0.662	1	114	regulation of cellular macromolecule biosynthetic process	BDEG_00252
GO:0008152	0.498	1.942	0.616	1	157	metabolic process	BDEG_08286
