# Supplementary material for: Genomic Correlates of Virulence Attenuation in the Deadly Amphibian Chytrid Fungus, Batrachochytrium dendrobatidis
Source: G3 (Bethesda). 2015 Sep 1;5(11):2291–8. doi: 10.1534/g3.115.021808 (PMC4632049; doi:10.1534/g3.115.021808)
Supplement: Supporting Information [file supp_g3.115.021808_TableS1.pdf]

Table S1 Summary of 2,231 nucleotide changes between two Bd isolates, JEL427-P9 and JEL427-P39, differing only in laboratory passage history (A). Following SNP filtering, summary information from the SNP dataset (e.g., genomic position, synonymous vs. nonsynonymous mutations) were extracted using snpEff software (version 2.0.5, Cingolani et al. 2012) and custom R scripts to compare the two isolates. The snpEff results were qualitatively similar to the pattern observed in the Rosenblum et al. 2013 dataset of 29 global isolates (B).

A: JEL427-P9 to JEL427-P39

Changes by type

	**# of changes**	**Per cent of changes**
**DOWNSTREAM**	894	25.88%
**INTERGENIC**	17	0.49%
**INTRON**	641	18.56%
**NON_SYNONYMOUS_CODING**	300	8.69%
**SPLICE_SITE_ACCEPTOR**	1	0.03%
**SPLICE_SITE_DONOR**	1	0.03%
**START_GAINED**	1	0.03%
**STOP_GAINED**	2	0.06%
**STOP_LOST**	0	0.00%
**SYNONYMOUS_CODING**	642	18.59%
**SYNONYMOUS_STOP**	2	0.06%
**UPSTREAM**	930	26.93%
**UTR_3_PRIME**	17	0.49%
**UTR_5_PRIME**	6	0.17%

**Chromosome**	**Length**	**Changes**	**Change rate**	**Change per kb**	**Normalized**
bden_JEL423_supercont1.1	4,440,149	503	8,827	0.113	0.051
bden_JEL423_supercont1.2	2,313,122	31	74,616	0.013	0.006

bden_JEL423_supercont1.3	1,829,408	62	29,506	0.034	0.015
bden_JEL423_supercont1.4	1,803,316	160	11,270	0.089	0.040
bden_JEL423_supercont1.5	1,707,251	42	40,648	0.025	0.011
bden_JEL423_supercont1.6	1,545,501	194	7,966	0.126	0.056
bden_JEL423_supercont1.7	1,398,854	263	5,318	0.188	0.084
bden_JEL423_supercont1.8	1,069,847	49	21,833	0.046	0.021
bden_JEL423_supercont1.9	1,057,463	123	8,597	0.116	0.052
bden_JEL423_supercont1.10	1,012,305	137	7,389	0.135	0.061
bden_JEL423_supercont1.11	979,369	58	16,885	0.059	0.027
bden_JEL423_supercont1.12	937,107	150	6,247	0.160	0.072
bden_JEL423_supercont1.13	898,261	158	5,685	0.176	0.079
bden_JEL423_supercont1.14	857,155	89	9,630	0.104	0.047
bden_JEL423_supercont1.15	557,602	100	5,576	0.179	0.080
bden_JEL423_supercont1.16	498,254	57	8,741	0.114	0.051
bden_JEL423_supercont1.17	243,426	50	4,868	0.205	0.092
bden_JEL423_supercont1.21	28,371	4	7,092	0.141	0.063
bden_JEL423_supercont1.46	7,014	1	7,014	0.143	0.064
**Total**	**23,183,77**	**2,231**	**10,391**	**0.096**	
**Ts/Tv ratio**	**5.4109**				

Genotype changes

	**#**	**proportion**
het -> hom	646	0.299
hom -> het	1477	0.683
hom1 -> hom2	38	0.018
**Total**	**2161**	

B: Results from Rosenblum *et al*. 2013 (Proc. Natl. Acad. Sci.)

Changes by type

	# of changes	Per cent of changes
**DOWNSTREAM**	49,685	26.59%
**INTERGENIC**	1,970	1.05%
**INTRON**	26,688	14.28%
**NON_SYNONYMOUS_CODING**	19,756	10.57%
**SPLICE_SITE_ACCEPTOR**	59	0.03%
**SPLICE_SITE_DONOR**	75	0.04%
**START_GAINED**	50	0.03%
**STOP_GAINED**	114	0.06%
**STOP_LOST**	26	0.01%
**SYNONYMOUS_CODING**	38,397	20.55%
**SYNONYMOUS_STOP**	51	0.03%
**UPSTREAM**	48,665	26.05%
**UTR_3_PRIME**	973	0.52%
**UTR_5_PRIME**	306	0.16%

Chromosome	Length	Changes	Change rate	Change per kb	Normalized
bden_JEL423_supercont1.1	4,440,149	23,541	188	5.302	0.043
bden_JEL423_supercont1.2	2,313,122	10,157	227	4.391	0.036
bden_JEL423_supercont1.3	1,829,408	9,588	190	5.241	0.043
bden_JEL423_supercont1.4	1,803,316	9,178	196	5.090	0.042
bden_JEL423_supercont1.5	1,707,251	8,741	195	5.120	0.042
bden_JEL423_supercont1.6	1,545,501	8,008	192	5.181	0.042
bden_JEL423_supercont1.7	1,398,854	7,764	180	5.550	0.045
bden_JEL423_supercont1.8	1,069,847	5,834	183	5.453	0.045
bden_JEL423_supercont1.9	1,057,463	6,080	173	5.750	0.047
bden_JEL423_supercont1.10	1,012,305	5,766	175	5.696	0.047
bden_JEL423_supercont1.11	979,369	5,427	180	5.541	0.045
bden_JEL423_supercont1.12	937,107	4,907	190	5.236	0.043

bden_JEL423_supercont1.13	898,261	5,433	165	6.048	0.049
bden_JEL423_supercont1.14	857,155	3,102	276	3.619	0.030
bden_JEL423_supercont1.15	557,602	4,124	135	7.396	0.061
bden_JEL423_supercont1.16	498,254	2,788	178	5.596	0.046
bden_JEL423_supercont1.17	243,426	1,333	182	5.476	0.045
bden_JEL423_supercont1.18	48,566	50	971	1.030	0.008
bden_JEL423_supercont1.19	69,118	118	585	1.707	0.014
bden_JEL423_supercont1.20	45,066	59	763	1.309	0.011
bden_JEL423_supercont1.21	28,371	222	127	7.825	0.064
**Total**	**23,339,511**	**122,220**		**5.237**	
