# Supplementary material for: Genomic Correlates of Virulence Attenuation in the Deadly Amphibian Chytrid Fungus, Batrachochytrium dendrobatidis
Source: G3 (Bethesda). 2015 Sep 1;5(11):2291–8. doi: 10.1534/g3.115.021808 (PMC4632049; doi:10.1534/g3.115.021808)
Supplement: Supporting Information [file supp_g3.115.021808_TableS2.pdf]

Table S2 Summary of potential functional relevance of nucleotide changes between two *Bd* isolates, JEL427-P9 and JEL427-P39, differing only in laboratory passage history. We assessed potential functional relevance of genes that had at least one nonsynonymous mutation in our dataset and were up-regulated on frog skin in Rosenblum *et al*. (2012) by conducting an over-representation test of Gene Ontology (GO) terms. We used the hypergeometric test in the GOstats R package (Falcon & Gentleman 2007) with the GO annotation assignments from Rosenblum *et al*. (2013). In all, 84 genes were both up-regulated and contained ≥1 nonsynonymous change. The hypergeometric test for over-representation of GO terms did not reveal any notable patterns, although the gene set contained one M36 metallopeptidase gene, a gene family that has previously been hypothesized to play a role in *Bd* virulence (Joneson *et al*. 2011).

GOBPID	P-value	Odds ratio	ExpCount	Count	Size	Term	Gene Ids
GO:0032313	0.006	21.595	0.116	2	20	regulation of Rab GTPase activity	BDEG_00189;BDEG_00594
GO:0006369	0.006	Inf	0.006	1	1	termination of RNA polymerase II transcription	BDEG_05286
GO:0043144	0.006	Inf	0.006	1	1	snoRNA processing	BDEG_05286
GO:0016180	0.006	Inf	0.006	1	1	snRNA processing	BDEG_05286
GO:0043087	0.009	16.870	0.145	2	25	regulation of GTPase activity	BDEG_00189;BDEG_00594
GO:0033121	0.009	16.870	0.145	2	25	regulation of purine nucleotide catabolic process	BDEG_00189;BDEG_00594
GO:0009118	0.009	16.870	0.145	2	25	regulation of nucleoside metabolic process	BDEG_00189;BDEG_00594
GO:0006140	0.009	16.161	0.151	2	26	regulation of nucleotide metabolic process	BDEG_00189;BDEG_00594
GO:0031329	0.009	16.161	0.151	2	26	regulation of cellular catabolic process	BDEG_00189;BDEG_00594
GO:0006821	0.012	182.533	0.012	1	2	chloride transport	BDEG_05538
GO:0048583	0.014	7.034	0.523	3	90	regulation of response to stimulus	BDEG_00189;BDEG_00452;BDEG_00594
GO:0010646	0.014	7.034	0.523	3	90	regulation of cell communication	BDEG_00189;BDEG_00452;BDEG_00594
GO:0023051	0.014	7.034	0.523	3	90	regulation of signaling	BDEG_00189;BDEG_00452;BDEG_00594
GO:0006303	0.017	91.233	0.017	1	3	double-strand break repair via nonhomologous end joining	BDEG_06507
GO:0016575	0.017	91.233	0.017	1	3	histone deacetylation	BDEG_00020
GO:0006012	0.017	91.233	0.017	1	3	galactose metabolic process	BDEG_04530
GO:0006807	0.018	3.247	4.565	9	786	nitrogen compound metabolic process	BDEG_00189;BDEG_00252;BDEG_00594;BDEG_03087; BDEG_03599;BDEG_04834;BDEG_05203;BDEG_05286;BDEG_06507
GO:0050790	0.027	8.957	0.261	2	45	regulation of catalytic activity	BDEG_00189;BDEG_00594
GO:0051174	0.027	8.957	0.261	2	45	regulation of phosphorus metabolic process	BDEG_00189;BDEG_00594
GO:0046578	0.032	8.182	0.285	2	49	regulation of Ras protein signal transduction	BDEG_00189;BDEG_00594
GO:0016569	0.034	36.453	0.035	1	6	covalent chromatin modification	BDEG_00020

GO:0035601	0.040	30.367	0.041	1	7	protein deacylation	BDEG_00020
GO:0052803	0.046	26.019	0.046	1	8	imidazole-containing compound metabolic process	BDEG_04834
GO:0000105	0.046	26.019	0.046	1	8	histidine biosynthetic process	BDEG_04834
GO:0006184	0.053	6.068	0.377	2	65	GTP catabolic process	BDEG_00189;BDEG_00594
GO:0006139	0.056	3.452	1.530	4	309	nucleobase-containing compound metabolic process	BDEG_03087;BDEG_03599;BDEG_05203;BDEG_06507
GO:0009146	0.056	5.877	0.389	2	67	purine nucleoside triphosphate catabolic process	BDEG_00189;BDEG_00594
GO:0009261	0.056	5.877	0.389	2	67	ribonucleotide catabolic process	BDEG_00189;BDEG_00594
GO:0009203	0.056	5.877	0.389	2	67	ribonucleoside triphosphate catabolic process	BDEG_00189;BDEG_00594
GO:0046130	0.056	5.877	0.389	2	67	purine ribonucleoside catabolic process	BDEG_00189;BDEG_00594
GO:0072523	0.056	5.877	0.389	2	67	purine-containing compound catabolic process	BDEG_00189;BDEG_00594
GO:0009164	0.058	5.786	0.395	2	68	nucleoside catabolic process	BDEG_00189;BDEG_00594
GO:1901292	0.062	5.528	0.412	2	71	nucleoside phosphate catabolic process	BDEG_00189;BDEG_00594
GO:1902531	0.062	5.528	0.412	2	71	regulation of intracellular signal transduction	BDEG_00189;BDEG_00594
GO:1901068	0.062	5.528	0.412	2	71	guanosine-containing compound metabolic process	BDEG_00189;BDEG_00594
GO:0065007	0.063	2.788	2.259	5	389	biological regulation	BDEG_00189;BDEG_00252;BDEG_00382;BDEG_00452; BDEG_00594
GO:0006357	0.084	12.976	0.087	1	15	regulation of transcription from RNA polymerase II promoter	BDEG_00252
GO:0006364	0.089	12.107	0.093	1	16	rRNA processing	BDEG_05286
GO:0009968	0.100	10.675	0.105	1	18	negative regulation of signal transduction	BDEG_00452
GO:0038032	0.100	10.675	0.105	1	18	termination of G-protein coupled receptor signaling pathway	BDEG_00452
GO:0008277	0.100	10.675	0.105	1	18	regulation of G-protein coupled receptor protein signaling pathway	BDEG_00452
GO:0009205	0.103	4.065	0.552	2	95	purine ribonucleoside triphosphate metabolic process	BDEG_00189;BDEG_00594
GO:0009141	0.105	4.020	0.558	2	96	nucleoside triphosphate metabolic process	BDEG_00189;BDEG_00594
GO:0006325	0.116	9.063	0.122	1	21	chromatin organization	BDEG_00020
GO:0042278	0.120	3.693	0.604	2	104	purine nucleoside metabolic process	BDEG_00189;BDEG_00594
GO:0005996	0.121	8.629	0.128	1	22	monosaccharide metabolic process	BDEG_04530
GO:0009150	0.128	3.549	0.627	2	108	purine ribonucleotide metabolic process	BDEG_00189;BDEG_00594
GO:0009119	0.130	3.514	0.633	2	109	ribonucleoside metabolic process	BDEG_00189;BDEG_00594
GO:0006820	0.131	7.872	0.139	1	24	anion transport	BDEG_05538
GO:0019693	0.132	3.480	0.639	2	110	ribose phosphate metabolic process	BDEG_00189;BDEG_00594
GO:0048523	0.141	7.237	0.151	1	26	negative regulation of cellular process	BDEG_00452
GO:0008033	0.146	6.956	0.157	1	27	tRNA processing	BDEG_05286

GO:1901657	0.148	3.230	0.685	2	118	glycosyl compound metabolic process	BDEG_00189;BDEG_00594
GO:0022613	0.176	5.640	0.192	1	33	ribonucleoprotein complex biogenesis	BDEG_05286
GO:0007166	0.190	5.150	0.209	1	36	cell surface receptor signaling pathway	BDEG_00452
GO:0071704	0.194	2.110	7.972	10	1481	organic substance metabolic process	BDEG_00020;BDEG_00252;BDEG_03087;BDEG_03599;BDEG_04530; BDEG_04834;BDEG_05033;BDEG_05203;BDEG_05286;BDEG_06507
GO:0071840	0.205	2.593	0.842	2	145	cellular component organization or biogenesis	BDEG_00020;BDEG_05286
GO:0006351	0.235	2.349	0.923	2	159	transcription, DNA-templated	BDEG_00252;BDEG_05286
GO:1902589	0.237	3.991	0.267	1	46	single-organism organelle organization	BDEG_00020
GO:0006281	0.241	3.903	0.273	1	47	DNA repair	BDEG_06507
GO:0033554	0.250	3.738	0.285	1	49	cellular response to stress	BDEG_06507
GO:0055086	0.261	2.172	0.993	2	171	nucleobase-containing small molecule metabolic process	BDEG_00189;BDEG_00594
GO:1901575	0.286	2.031	1.057	2	182	organic substance catabolic process	BDEG_00189;BDEG_00594
GO:0051716	0.286	2.037	1.059	2	191	cellular response to stimulus	BDEG_00382;BDEG_00452
GO:0023052	0.306	1.927	1.109	2	191	signaling	BDEG_00382;BDEG_00452
GO:0008652	0.314	2.832	0.372	1	64	cellular amino acid biosynthetic process	BDEG_04834
GO:0044237	0.322	1.449	8.566	10	1475	cellular metabolic process	BDEG_00020;BDEG_00189;BDEG_00252;BDEG_00594;BDEG_03087; BDEG_03599;BDEG_04834;BDEG_05203;BDEG_05286;BDEG_06507
GO:1901605	0.326	2.700	0.389	1	67	alpha-amino acid metabolic process	BDEG_04834
GO:0034660	0.334	2.619	0.401	1	69	ncRNA metabolic process	BDEG_05286
GO:0035556	0.347	2.497	0.419	1	71	intracellular signal transduction	BDEG_00382
GO:0044710	0.377	1.502	2.183	3	451	single-organism metabolic process	BDEG_00020;BDEG_04530;BDEG_04834
GO:0050794	0.385	1.619	1.316	2	269	regulation of cellular process	BDEG_00252;BDEG_00382
GO:0034654	0.391	1.573	1.336	2	230	nucleobase-containing compound biosynthetic process	BDEG_00252;BDEG_05286
GO:0016053	0.399	2.082	0.499	1	86	organic acid biosynthetic process	BDEG_04834
GO:0006259	0.429	1.891	0.548	1	99	DNA metabolic process	BDEG_03087
GO:0044763	0.434	1.311	3.316	4	689	single-organism cellular process	BDEG_00382;BDEG_00452;BDEG_04834;BDEG_05538
GO:0019219	0.439	1.839	0.564	1	110	regulation of nucleobase-containing compound metabolic process	BDEG_00252
GO:2001141	0.474	1.640	0.627	1	108	regulation of RNA biosynthetic process	BDEG_00252
GO:0006396	0.477	1.624	0.633	1	109	RNA processing	BDEG_05286
GO:0006629	0.486	1.578	0.650	1	112	lipid metabolic process	BDEG_00382
GO:0009889	0.492	1.549	0.662	1	114	regulation of biosynthetic process	BDEG_00252
GO:2000112	0.492	1.549	0.662	1	114	regulation of cellular macromolecule biosynthetic process	BDEG_00252
GO:0008152	0.498	1.942	0.616	1	157	metabolic process	BDEG_08286
